# Cognitive function in dyslipidemia patients: exploring the impact of statins

**DOI:** 10.3389/fneur.2024.1436010

**Published:** 2024-09-16

**Authors:** Wenxiao Wang, Xin Li

**Affiliations:** ^1^State Key Laboratory of Cognitive Neuroscience and Learning, Beijing Normal University, Beijing, China; ^2^School of System Science, Beijing Normal University, Beijing, China; ^3^BABRI Centre, Beijing Normal University, Beijing, China

**Keywords:** cognitive function, statins, dyslipidemia, aging cohorts, middle-aged, elderly

## Abstract

**Background:**

Evidence regarding the relationship between the use of statins and cognitive outcomes presents varying findings. This study aims to analyze the relationship between sustained statin use and cognitive performance in dyslipidemia patients.

**Methods:**

This study presents findings from the Beijing Ageing Brain Rejuvenation Initiative (BABRI) study, in which a cohort of community-dwelling dyslipidemia patients (Entire sample, *N* = 1,062, aged 50–86) was recruited. Participants were divided into two groups based on their sustained use statins (Statins group, *N* = 677) or not use any lipid-lowering agents (Untreated group, *N* = 385). Furthermore, the entire sample was stratified by age into the middle-aged sample (*N* = 451) and the older people sample (*N* = 611), following a similar categorization based on statin application. ANCOVA was used to evaluate the relationship between sustained statin use and cognitive function.

**Results:**

Overall, in the total sample, the statins group demonstrated better cognition in overall cognition, memory, visuospatial ability, attention, executive function, and language domains compared to the untreated group. Moreover, the statins group only showed better performance in attention among the middle-aged sample. In the older people sample, statins group exhibited superior cognitive performance across various cognitive domains compared to untreated group.

**Conclusion:**

Among dyslipidemia patients in Beijing community, sustained statin users exhibited superior cognitive function across all domains compared to untreated individuals, with particularly noticeable improvements among those aged 65 and above. These findings underscore the protective effect of statins on cognitive function in dyslipidemia patients, highlighting significant benefits for the older people population.

## 1 Introduction

Statins, commonly prescribed for cardiovascular conditions such as dyslipidemia and etc., primarily act by inhibiting 3-hydroxy-3-methylglutaryl-coenzyme A (HMG-CoA) reductase, thereby reducing cholesterol synthesis (1). Given the established link between elevated cholesterol and cardiovascular diseases, statins play a pivotal role in lowering peripheral blood cholesterol, particularly in reducing low-density lipoprotein cholesterol (LDL-C), thereby aiding in the reduction of atherosclerotic cardiovascular disease and other cardiovascular risks (2, 3). Beyond their established role in managing lipid metabolism and serving as primary prevention or secondary prevention for coronary heart disease and cerebrovascular diseases, research suggests that statins may offer potential benefits in reduce the risk of cognitive impairment-associated conditions such as Alzheimer’s disease (AD) and dementia (4, 5). The observed effect could be attributed to the ability of statins to penetrate the blood–brain barrier (BBB) (6, 7), along with their anti-inflammatory, antioxidant, and synaptic plasticity-regulating properties (8, 9). Moreover, they regulate cerebral cholesterol metabolism, thereby promoting neuroprotection (10, 11). At the same time, other studies have found that statins can regulate hippocampal neurogenesis by upregulating Wnt signaling through dependent inhibition of the Mevalonate (MVA) Pathway (12). Additionally, there is evidence that statins are effective inducers of axon and dendrite growth (13). In summary, the regulation of cognitive function by statins may involve two key mechanisms: first, by modulating cholesterol metabolism and other pathways in the central nervous system, and second, by lowering peripheral blood cholesterol to reduce cardiovascular and cerebrovascular risks (14). These multifaceted effects provide potential biological explanations for the effects of statins on cognitive function. Despite these benefits, controversy remains surrounding the relationship between statin use and cognition in the general population. On February 28, 2012, the U.S. Food and Drug Administration (FDA) mandated the inclusion of a warning label on statins, due to insights provided from post-marketing surveillance, observational studies (15–19), and randomized controlled trials (RCTs) (20, 21) hinting at potential adverse effects on cognitive function. Critiques of RCTs and observational data suggest a potential link between this adverse effect and the use of high-dose statins (22). A review summarizing a series of randomized controlled trials found that statins did not exhibit clear adverse effects on patients’ cognitive function in short-term studies. However, long-term follow-up research indicated a significant reduction in the incidence of dementia among patients treated with statins (23). However, there is still no conclusive evidence that statins cause clinically significant cognitive impairment (22, 24, 25) or that statins reduce the risk of dementia or cognitive impairment (26).

Based on the above background, we decided to use data from the Beijing Aged Brain Rejuvenation Initiative (BABRI) cohort, which is a community-based cohort study that mainly focuses on population aging, especially cognitive aging and its determinants. A screening of 1,062 dyslipidemia patients was conducted using the baseline database of the BABRI cohort. The primary objective was to assess sustained use of statin drugs affect various cognitive functions in dyslipidemia patients compared to those not using any lipid-lowering medication. The aim was to precisely pinpoint the impact of statins on various cognitive functions in individuals dealing with dyslipidemia. Subsequently, dyslipidemia patients were categorized into two sample—older people and middle-aged—in order to investigate more comprehensively the effects of statins on cognitive function in hyperlipidemic patients across different age groups.

## 2 Materials and methods

### 2.1 Study cohort and measures

Participants in the cross-sectional study were sourced from the Beijing community, a prospective, community-based cohort (27, 28). This study selected a group of dyslipidemia patients from the BABRI baseline database as follows. Among the 7,625 participants included in the BABRI database and meeting the specified inclusion and exclusion criteria, a group consisting of 1,477 individuals with dyslipidemia was identified. The inclusion criteria as follows: (1) individuals aged 50 or above; (2) attainment of 6 or more years of formal education; (3) all diagnoses of dyslipidemia were made by physicians in Beijing area’s tertiary hospitals, according to the 2018 AHA Guideline on the Management of Blood Cholesterol (29), validated by medical records from community healthcare centers; (4) willingness to engage in face-to-face interviews. Exclusion criteria are as follows: (1) individuals diagnosed with dementia, Parkinson’s disease, other degenerative neurological disorders, psychiatric conditions, or brain tumors; (2) incapacity to undergo cognitive assessments due to physical or mental disabilities.

Furthermore, the medication status of dyslipidemia patients was determined through their medical records or self-reports, with exclusive attention given to the utilization of any medication falling within the class of HMG-CoA reductase inhibitors (Including any hydrophilic or lipophilic statins, with no dose restrictions, such as simvastatin, atorvastatin, rosuvastatin, etc.), with a minimum duration of continuous usage exceeding 6 months. Following the exclusion of patients with unclear or irregular medication records and those taking alternative lipidlowering agents, a final sample of 1,062 dyslipidemia patients with well-documented medication records were categorized into a statins-user group (*n* = 677) and an untreated group (*n* = 385).

Other covariates encompassed age, gender, education, diabetes, hypertension, smoking and obesity. Face-to-face interviews were used to assess age, education, gender and smoking and obesity. Smoking status was determined by self-reported smoking habits. Participants were classified as obese if their body mass index (BMI) exceeded ≥30, based on criteria from the World Health Organization Global Health Observatory data. Additionally, in accordance with guidelines from the ADA and AHA, diagnoses of type 2 diabetes and hypertension were performed by physicians at tertiary hospitals in the Beijing area, with patient medical records reviewed at community health care centers.

### 2.2 Neuropsychological tests

The current study employed a comprehensive neuropsychological test battery to evaluate general cognition and five cognitive domains, consistent with previous research (27). The Mini-Mental State Examination (MMSE) (30) and Montreal Cognitive Assessment (MoCA) (31) acted as comprehensive tools to measure general cognitive function. Memory assessment involved the Auditory Verbal Learning Test (AVLT) (32) and recall in the Rey-Osterrieth Complex Figure Test (CFT) (33). Evaluating visuospatial ability involved administering the CFT copy and the Clock-Drawing Test (CDT) (34). Language proficiency was gauged using the Category Verbal Fluency Test (CVFT) (35) and the Boston Naming Test (BNT) (36). Attention was scrutinized via the Trail-Making Test (TMT) (37) part A and the Symbol Digit Modalities Tests (SDMT) (38), while executive function was measured by the TMT part B and the Stroop Color-Word Test (SCWT) (39).

### 2.3 Statistical analysis

Demographic characteristics, cognitive performance, and disease status were reported separately for the total sample (*n* = 1,062), the middle-aged sample (*n* = 451), and the older people sample (*n* = 611). One-way ANOVA or the *χ*^2^ test was used to test for significant differences between the groups. Given the known impact of age on cognitive function, participants were further categorized into middle-aged and older people sub sample. In these sub sample, the effects of regular statins use on each cognitive test was assessed using one-way ANCOVA, with age, gender, education, hypertension, and diabetes as concomitant variables. All analyses were performed in SPSS 27.0 (IBM Corp, Armonk, NY).

## 3 Results

**Figure 1:**
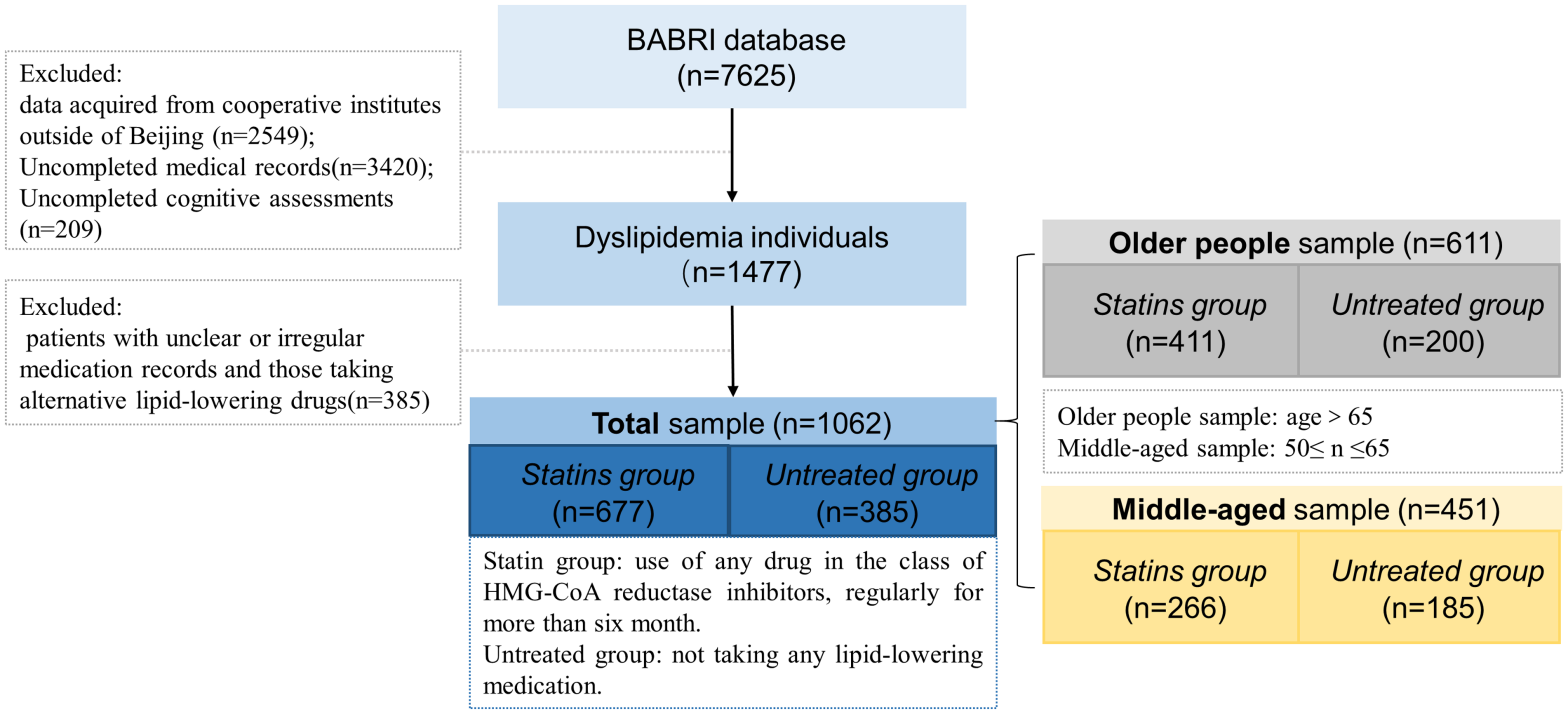
Flow chart of the Dyslipidemia patients inclusion process. The total data volume from the BABRI database (*n* = 7,625) presented in this flowchart is accurate up to January 2018. Since that time, the database has continually expanded, and BABRI has now enrolled over 10,000 participants.

Among the 7,625 participants screened in the BABRI database, participants from non-Beijing communities (*n* = 2,549) were first excluded, as well as those with incomplete basic medical records (*n* = 3,420) and incomplete cognitive assessments (*n* = 209). Based on this criterion, a total of 1,477 patients with dyslipidemia were identified. Of these, 385 patients were excluded due to unclear or non-standardized treatment records. Among the remaining 1,062 patients, 677 regularly used statins for more than 6 months (Statins group), while 385 patients did not receive any intervention (Untreated group). To investigate the potential benefits of statin usage across various age groups, the 1,062 patients were further divided into a middle-aged sample (*n* = 611) and an older people sample (*n* = 451). Within these two samples, the middle-aged statin group comprised 266 individuals, with the middle-aged untreated group consisting of 185 individuals. The older people statin group encompassed 411 individuals, whereas the older people untreated group comprised 200 individuals (see Figure 1).

Among the total sample of 1,062 participants with dyslipidemia, the average age was 65.91 ± 7.37 years. The statins group, with an average age of 66.46 ± 7.33 years, was older than the untreated group (64.95 ± 7.38 years, *F* = 10.4, *p* = 0.001). Education (*F* = 2.05, *p* = 0.15) and gender (*χ*^2^ = 0.02, *p* = 0.889) showed no significant differences between the two groups (Table 1). In the middle-aged sample, the statins group (59.21 ± 3.52 years) and untreated group (58.81 ± 3.73 years) exhibited no significant differences in age (*F* = 1.34, *p* = 0.25), education (*F* = 1.17, *p* = 0.28), and gender (*χ*^2^ = 1, *p* = 0.49) (Table 2). Within the older people sample (average age 76.4 ± 6.7 years), the statins group (71.16 ± 4.95 years) and untreated group (70.63 ± 4.97 years) showed no significant differences in age (*F* = 1.53, *p* = 0.21), education (*F* = 1.22, *p* = 0.27), or gender (*χ*^2^ = 0.52, *p* = 0.29) (Table 3). Simultaneously, both in the total sample and within the middle-aged and older people sub sample, the prevalence of hypertension and diabetes was significantly higher in the statins group in comparison to the untreated group.

**Table 1:** Significant intergroup differences in demographic data and multidomain cognitive performance of the two groups in total patients.

Variables (M ± SD)	Untreated (*n* = 385)	Statins (*n* = 677)	F/*χ*^2^	*P*
Demographic information				
Age	64.95 ± 7.38	66.46 ± 7.33	10.4	0.001**
Education	11.18 ± 2.68	11.43 ± 2.76	2.05	0.15
Gender	116/269	201/476	0.02	0.889
Physical health				
Type 2 Diabetes	107/278	283/394	20.73	<0.001***
Hypertension	224/161	476/201	16.07	<0.001***
Smoking	46/339	62/615	2.25	0.134
Obesity	35/350	49/628	1.16	0.218
General cognition				
MMSE	27.45 ± 2.18	27.56 ± 2.11	2.46	0.117
MOCA	22.34 ± 2.51	22.83 ± 3.23	9.96	0.002**
Memory				
AVLT-N5	4.93 ± 2.48	4.89 ± 2.40	0.19	0.660
AVLT-N1N5	27.23 ± 8.43	27.23 ± 8.41	0.53	0.466
CFT delay	12.33 ± 7.30	13.04 ± 6.63	4.6	0.032*
Visuospatial ability				
CFT copy	32.97 ± 5.39	33.71 ± 3.92	8.64	0.003**
CDT	23.18 ± 5.45	23.46 ± 5.49	0.53	0.468
Attention				
SDMT	33.05 ± 10.39	33.10 ± 10.07	2.47	0.117
TMTA	58.96 ± 22.13	60.04 ± 22.39	0.07	0.796
DST	12.01 ± 2.11	12.41 ± 2.14	14.71	<0.001***
Executive function				
SCWT	78.47 ± 22.22	78.48 ± 21.93	1.82	0.178
TMTB	170.58 ± 68.92	165.97 ± 65.87	7.59	0.006**
Language				
VFT	45.55 ± 8.71	45.42 ± 8.72	0.47	0.492
BNT	23.48 ± 3.48	24.09 ± 3.27	11.23	0.001**

Values are mean ± SD or Nos. of participants. The comparisons of age, education, and various cognitive function between the two groups were performed with ANOVA. The *p*-values for gender, diabetes, hypertension, obesity, and smoking ratio were obtained using a Chi-square test. Significance: *p < 0.05, **p < 0.01, ***p < 0.001. Edu, Education; MMSE, Mini-mental state examination; MoCA, Montreal cognitive assessment; AVLT-N5, Auditory verbal learning test number 5; AVLT-N1N5, Auditory verbal learning test number1-number5; CFT-delay, Recall in the Rey-Osterrieth complex figure test; CFT copy, Copy in the Rey-Osterrieth complex; CDT, Clock-drawing test; SDMT, Symbol digit modalities tests; TMTA, Trail-making test part A; DST, Digital symbol test; SCWT, Stroop color-word test; TMTB, Trail-making test part B; CVFT, Category verbal fluency test; BNT, Boston naming test.

**Table 2:** Significant intergroup differences in demographic data and multi domain cognitive performance of the two groups in older people sample.

Variables (M ± SD)	Untreated (*n* = 200)	Statins (*n* = 411)	*F*/*χ*^2^	*P*
Demographic information				
Age	70.63 ± 4.97	71.16 ± 4.95	1.53	0.219
Education	11.09 ± 3.08	11.37 ± 3.02	1.22	0.269
Gender	71/129	135/276	0.52	0.287
Physical health				
Type 2 Diabetes	52/148	180/231	18.09	<0.001***
Hypertension	125/75	302/109	7.71	0.006**
Smoking	20/180	30/381	1.48	0.225
Obesity	16/184	20/391	1.96	0.161
General cognition				
MMSE	27.21 ± 2.33	27.39 ± 2.16	1.25	0.265
MOCA	22.27 ± 3.75	22.81 ± 3.16	8.76	0.003**
Memory				
N5	4.61 ± 2.52	4.59 ± 2.47	0.04	0.842
N1N5	27.95 ± 8.29	26.14 ± 8.57	0.27	0.605
CFT delay	11.58 ± 7.04	12.76 ± 6.62	6.61	0.014*
Visuospatial ability				
CFT copy	32.59 ± 6.07	33.50 ± 4.31	5.72	0.017*
CDT	23.79 ± 5.37	23.99 ± 5.55	0.89	0.345
Attention				
SDMT	30.61 ± 9.72	30.88 ± 9.8	1.23	0.268
TMTA	63.12 ± 22.83	63.66 ± 23.46	3.82	0.051
DST	11.82 ± 2.19	12.24 ± 2.23	9.33	0.002**
Executive function				
SCWT	85.22 ± 24.32	82.19 ± 22.18	4.53	0.034*
TMTB	189.34 ± 75.66	180.33 ± 73.18	5.75	0.017*
Language				
VFT	43.75 ± 8.54	44.11 ± 8.86	0.52	0.473
BNT	23.36 ± 3.59	24.08 ± 3.48	9.45	0.002**

Values are mean ± SD or Nos. of participants. The comparisons of age, education, and various cognitive function between the two groups were performed with ANOVA. The *p*-values for gender, diabetes, hypertension, obesity, and smoking ratio were obtained using a Chi-square test. Significance: *p < 0.05, **p < 0.01, ***p < 0.001 MMSE, Mini-mental state examination; MoCA, Montreal cognitive assessment; AVLT-N5, Auditory verbal learning test number 5; AVLT-N1N5, Auditory verbal learning test number1-number5; CFT-delay, Recall in the Rey-Osterrieth complex figure test; CFT copy, Copy in the Rey-Osterrieth complex; CDT, Clock-drawing test; SDMT, Symbol digit modalities tests; TMTA, Trail-making test part A; DST, Digital symbol test; SCWT, Stroop color-word test; TMTB, Trail-making test part B; CVFT, Category verbal fluency test; BNT, Boston naming test.

**Table 3:** Significant intergroup differences in demographic data and multidomain cognitive performance of the two groups in middle age patients.

Variables (M ± SD)	Untreated (*n* = 185)	Statins (*n* = 266)	F/*χ*^2^	*P*
Demographic information				
Age	58.81 ± 3.73	59.21 ± 3.52	1.34	0.247
Education	11.29 ± 2.16	11.52 ± 2.3	1.17	0.280
Gender	45/140	66/200	1	0.499
Physical health				
Type 2 Diabetes	55/130	103/153	3.88	0.049*
Hypertension	99/86	174/92	6.47	0.011*
Smoking	26/159	32/234	0.45	0.505
Obesity	19/166	27/239	0.02	0.898
General cognition				
MMSE	27.71 ± 1.99	27.82 ± 1.87	0.73	0.392
MOCA	22.41 ± 3.256	22.86 ± 2.887	1.91	0.168
Memory				
N5	5.25 ± 2.394	5.35 ± 2.224	0.02	0.889
N1N5	28.57 ± 8.38	28.92 ± 7.886	0.03	0.874
CFT delay	13.11 ± 7.511	13.46 ± 6.64	0.07	0.789
Visuospatial ability				
CFT copy	33.36 ± 4.547	34.03 ± 3.18	3.04	0.082
CDT	22.52 ± 5.48	22.63 ± 5.356	0.01	0.935
Attention				
SDMT	35.57 ± 10.479	36.48 ± 9.529	1.05	0.306
TMTA	54.59 ± 20.543	54.45 ± 19.815	0.01	0.942
DST	12.22 ± 1.996	12.66 ± 1.96	5.49	0.020*
Executive function				
SCWT	71.42 ± 17.133	72.94 ± 20.376	0.37	0.545
TMTB	150.98 ± 54.80	143.96 ± 44.639	1.71	0.191
Language				
VFT	47.43 ± 8.51	47.47 ± 8.08	0.01	0.970
BNT	23.60 ± 3.372	24.11 ± 3.051	1.81	0.179

Values are mean ± SD or Nos. of participants. The comparisons of age, education, and various cognitive function between the two groups were performed with ANOVA. The *p*-values for gender, diabetes, hypertension, obesity, and smoking ratio were obtained using a Chi-square test. Significance: *p < 0.05, **p < 0.01, ***p < 0.001. Edu, Education; MMSE, Mini-mental state examination; MoCA, Montreal cognitive assessment; AVLT-N5, Auditory verbal learning test number 5; AVLT-N1N5, Auditory verbal learning test number1-number5; CFT-delay, Recall in the Rey-Osterrieth complex figure test; CFT copy, Copy in the Rey-Osterrieth complex; CDT, Clock-drawing test; SDMT, Symbol digit modalities tests; TMTA, Trail-making test part A; DST, Digital symbol test; SCWT, Stroop color-word test; TMTB, Trail-making test part B; CVFT, Category verbal fluency test; BNT, Boston naming test.

In the comprehensive sample of 1,062 patients, significant differences were observed between the statin group and the untreated group across multiple cognitive domains, including general cognitive function (MoCA, *F* = 9.96, *p* = 0.002), memory (CFT delay, *F* = 4.6, *p* = 0.032), visual–spatial function (CFT copy, *F* = 8.64, *p* = 0.003), attention (DST, *F* = 14.71, *p* < 0.001), executive function (TMTB, *F* = 7.588, *p* = 0.006), and language (BNT, *F* = 11.23, *p* = 0.001) (see Table 1). In the older people sample, the statin group exhibited differences compared to the untreated group across general cognitive function (MoCA, *F* = 8.76, p = 0.003), memory (CFT delay, *F* = 6.61, *p* = 0.014), visual–spatial function (CFT copy, *F* = 5.72, *p* = 0.017), attention (DST, *F* = 9.33, *p* = 0.002), executive function (SCWT, *F* = 4.5, *p* = 0.034; TMTB, *F* = 5.75, p = 0.017), and language (BNT, *F* = 9.445, p = 0.002) (see Table 2). Finally, in the middle-aged sample, the statin group exhibited differences only in the attention domain (DST, *F* = 5.488, *p* = 0.02) (see Table 3).

## 4 Discussion

Our population-based study unveiled a significant correlation between sustained statin use and enhanced cognitive performance among dyslipidemia patients in Beijing communities. Even after adjusting for demographic variables and potential confounders, such as other cardiovascular risks, dyslipidemia patients who regularly took statins demonstrated notably improved cognitive performance, particularly in executive function, memory, and language. Furthermore, this trend persisted across both middle-aged and older people samples, albeit with a slight decrease observed in the middle-aged group and a more pronounced effect in individuals aged 65 and above.

The lipid peroxidation theory of dementia suggests that damage to the BBB in dementia patients leads to the entry of external lipids into the brain, resulting in the accumulation of “adipose inclusion” and abnormalities in brain lipid metabolism, brain cholesterol alters the degradation of amyloid precursor protein, triggering the onset of dementia and accelerating the progression of dementia (40, 41). Consequently, dyslipidemia is thought to be an important risk factor for cognitive dysfunction and dementia (42, 43). Cognitive impairment is frequently observed to be accompanied by elevated serum cholesterol and low-density lipoprotein (LDL) levels (44). Even after adjusting for factors such as age and the APOEε4 allele, an increase in serum cholesterol remains associated with a threefold increase in dementia risk (45). It is commonly believed that lipid-lowering therapy is thought to be beneficial in reducing the incidence of AD and delaying cognitive decline (46). Our study emphasizes that regular use of statin medications has a positive impact on the cognitive function of patients with dyslipidemia. In addition, the beneficial effect is more pronounced in individuals aged 65 and above, especially among those who are already susceptible to cognitive impairment. These results are in line with previous observational cohort studies (16, 47), whether employed for cholesterol regulation in dyslipidemia patients or as a preventive measure against various cardiovascular and cerebrovascular conditions (48), statins have demonstrated a favorable influence on cognitive function. The explanation for differences across older people and middle-aged samples can be attributed to the fact that older people dyslipidemia participants, who are more likely to use statin medications over an extended period and often have a higher prevalence of chronic conditions such as hypertension and diabetes, may benefit from the potential accumulation of long-term protective effects and the mitigation of cognitive decline by controlling underlying risk factors, as well as from potential neuroprotective effects due to their aging nervous system. The differences across different age groups have also been confirmed in previous studies, indicating that statin use can reduce the incidence of dementia in healthy older people populations (49). Certainly, dietary therapy is also an important treatment method for patients with dyslipidemia (50). In this study, all patients received dietary therapy guidance and advice from hospitals, community health service centers, and our team. However, since the majority of the dyslipidemia patients included in this study are from northern China, where dietary habits tend to be rich in oil, salt, sugar, and fat, it is challenging for them to adhere to a healthy dietary regimen. Therefore, for most patients, taking medication regularly is easier than maintaining a healthy diet.

Additionally, it is worth noting that this study primarily examines the benefits of sustained statin use in community-dwelling patients with dyslipidemia, with a focus on a population that is not comprised of cognitive impairment or dementia patients. Therefore, it does not address potential time-dependent confounding factors related to worsened medication adherence due to cognitive impairment (51). Of course, there are conflicting views in epidemiological studies regarding the impact of statin drugs on dementia (51, 52), particularly in randomized controlled trials (RCTs) where the use of statin drugs has been shown not to reduce the risk of dementia (53, 54). Currently, there is no consensus on the potential efficacy of statins in preventing dementia or AD (22, 51). Padala et al. (55, 56) found in their studies on populations with dementia and cognitive impairment that statin use can lead to cognitive decline, while discontinuing statins may result in the reversal of cognitive impairment. In contrast, the Rotterdam Study found that statin use, whether lipophilic or hydrophilic, was associated with a reduced risk of Alzheimer’s disease in the general population (57). An absence of a consensus may stem from differences in experimental design across studies, such as the selection of study populations. Patients who already have cognitive impairments may respond differently to medication compared to those with normal cognition. This is particularly relevant for lipophilic statins, which may regulate cholesterol production in the brain, affecting neuronal structure and function and leading to transient cognitive decline (69). Additionally, factors such as study duration, medication dosage and concurrent drug use, the cognitive assessment tools used, and potential biases introduced by different study populations may also contribute to the variability in results. However, patients who accept and continue statin therapy are significantly associated with their education level, socioeconomic status, and cholesterol levels, all of which are closely related to the risk of dementia. Since the participants in this study were all older individuals from urban areas, this group tends to have better health awareness and medication adherence compared to older individuals from rural areas, this “healthy user effect” is also one of the issues that this study needs to address (58).

Our study has several strengths. Firstly, it explores the relationship between statin medication use and cognitive function in a relatively large community population, reflecting real-world conditions. Additionally, the study provides a comprehensive assessment of multidimensional cognitive function in all dyslipidemia patients. The findings indicate that the statins group outperformed the untreated group in various cognitive domains. Previous research has often been limited by focusing solely on employing one or two cognitive tests (47, 53), while our study comprehensively assessed the impact of statins on cognitive function in various domains among dyslipidemia patients. Furthermore, this study also investigated the relationship between statin medications and cognitive function in patients with dyslipidemia across different age groups. The findings revealed that the benefits of regular statin use are more significant in the older people sample, while in the middle-aged group, cognitive function gains from regular statin use are only evident as a trend.

While this study provides valuable insights, it is not without limitations. First, as a cross-sectional study, it cannot determine the long-term effects of statins on individuals with dyslipidemia. Longitudinal follow-up studies or well-designed randomized controlled trials are needed to optimize experimental design and analysis methods. These studies should precisely account for various confounding variables, including lipid fluctuations (59), different genetic variations (such as APOE, LDLR, CETP, etc.) (60, 61), guidelines for treatment of dyslipidemia, statin dosage (62, 63), and sex differences (64), to minimize bias and accurately quantify the specific cognitive benefits of statins for individuals with dyslipidemia. Additionally, this study solely explores the impact of statin therapy on the cognitive function of dyslipidemia patients and does not include individuals at cardiovascular risk who use statins for preventive purposes. Due to constraints in acquiring biological specimens, this study was not able to account for the APOE ε4 carriage status among subjects. Recent research suggests that the advantageous cognitive effects of statin therapy might be more pronounced among carriers of the APOE ε4 allele (65). Additionally, it is important to note that due to limitations in the available data, our study did not differentiate between the hydrophilic and lipophilic properties of statin medications among participants. While prior research has explored this issue, consensus on whether different types of statins exhibit divergent effects on cognition remains inconclusive (23, 66). Despite lipophilic statins being more likely to enter the central nervous system compared to hydrophilic statins, hydrophilic statins can also cross the blood–brain barrier with the assistance of certain active transport proteins, such as the OATP family transporters (67, 68). The impact of these confounding factors may constrain inferences drawn from observational studies, leading to conclusions that could vary to some extent based on the specific cohorts examined and the potential confounding variables controlled for in multivariate analyses.

## 5 Conclusion

Our population-based study has unveiled a notable correlation between consistent statin usage and improved cognitive performance among dyslipidemia patients residing in Beijing communities. Even after adjusting for demographic variables and potential confounders, such as other cardiovascular risks, dyslipidemia patients who maintained regular statin intake exhibited enhanced cognitive performance, notably in executive function, memory, and language. Moreover, this effect remained consistent across both middle-aged and older people samples, although a slight decrease was observed in the middle-aged group compared to a more pronounced impact in individuals aged 65 and above. Looking ahead, there is a pressing need for long-term follow-up studies or meticulously designed randomized controlled trials to comprehensively understand and quantify the specific cognitive benefits conferred by statin medications in dyslipidemia patients.

## Data Availability

The original contributions presented in the study are included in the article/supplementary material, further inquiries can be directed to the corresponding author.
